# Supplementary material for: Divergent community assembly processes and multifunctionality contributions of abundant and rare soil bacteria during a 53-year restoration in the Tengger Desert, China
Source: Commun Biol. 2025 Sep 26;8:1376. doi: 10.1038/s42003-025-08764-8 (PMC12474879; doi:10.1038/s42003-025-08764-8)
Supplement: Supplementary file 1 — Supplementary information [file 42003_2025_8764_MOESM1_ESM.pdf]

Supplementary information for

Divergent community assembly processes and multifunctionality contributions of abundant and rare soil bacteria during a 53-year restoration in the Tengger Desert, China

Qingqing Hou[1,4], Rui Xia[1,4], Bodong Yuan[1], Muhammad Aqeel[1], Ying Sun[1], Longwei Dong[1], Abdul Manan[1], Fan Li[1], Yan Deng[1], Xusheng Guo[2], Guili Wu[1], Jinzhi Ran[1], Weigang Hu[1]*, Jihua Wu[1], Xinrong Li[3], Jianming Deng[1]*

[1]State Key Laboratory of Herbage Improvement and Grassland Agro-ecosystems, College of Ecology, Lanzhou University, Lanzhou 730000, China.

[2]School of Life Sciences, Lanzhou University, Lanzhou, 730000, China

[3]Shapotou Desert Research and Experiment Station, Northwest Institute of Eco-Environment and Resources, Chinese Academy of Sciences, Lanzhou 730000, China

[4]These authors contributed equally: Qingqing Hou, Rui Xia

*Corresponding author: huweigang@lzu.edu.cn (Weigang Hu); dengjm@lzu.edu.cn (Jianming Deng)

This PDF file includes:

Supplementary Figs. 1–16

Supplementary Table 1–2

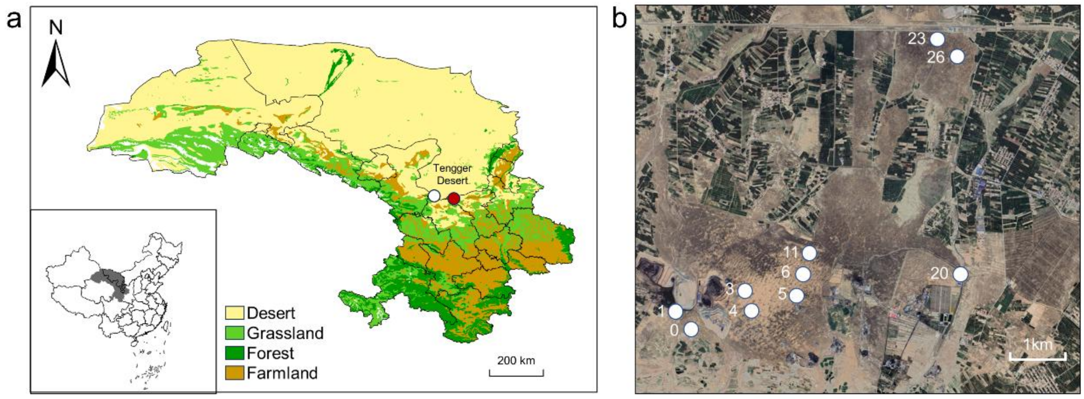

Supplementary Figure 1. Maps of study area and plots with different restoration durations. a The study area is located on the southern edge of the Tengger Desert in northwest China. White circle indicates the main sampling area of Jingtai County; red circle represents the 53-year restoration plot in the Shapotou restoration area. **b** White circles and numbers indicate plots with different restoration duration (years) within the main sampling area. It should be noted that the 53-year restoration plot is geographically separated from these plots.

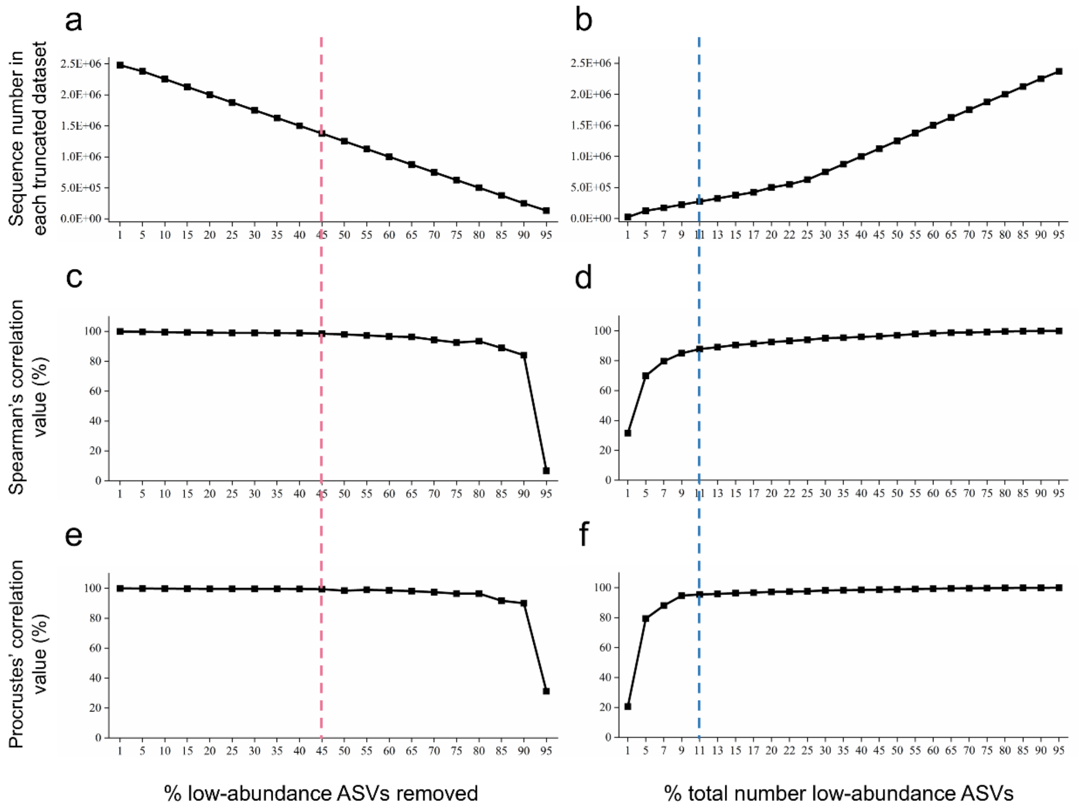

Supplementary Figure 2. MultiCoLA profiles for dataset structure and most important axes of extracted variation based on the dataset-based cutoff approach. **a**, **c**, **e** The rare ASVs were removed. **b**, **d**, **f** The rare ASVs were retained. **a**, **b** The sequence number in each truncated dataset. **c**, **d** Non-parametric Spearman correlations comparing the deviation in complete data structure between the original and truncated matrices. **e**, **f** Procrustes method comparing most important axes of extracted variation from original versus truncated matrices via NMDS. The pink and blue dashed lines indicated the thresholds of abundant and rare ASVs in this study, respectively.

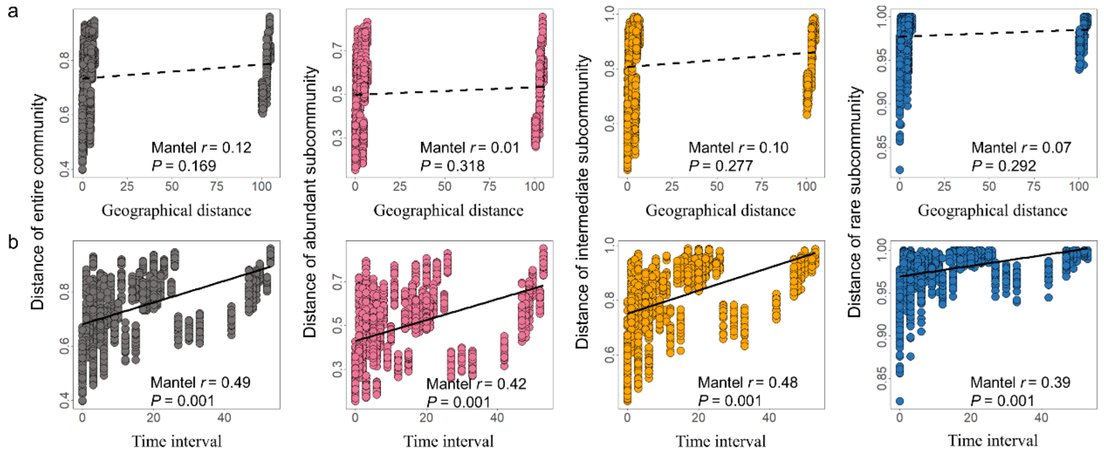

Supplementary Figure 3. Relationships between the differences in bacterial community composition and geographical distance and time interval. a Geographical distance; **b** Time interval. The solid and dotted lines represent statistically significant ($P \leq 0.05$) and nonsignificant ($P > 0.05$) relationships, respectively.

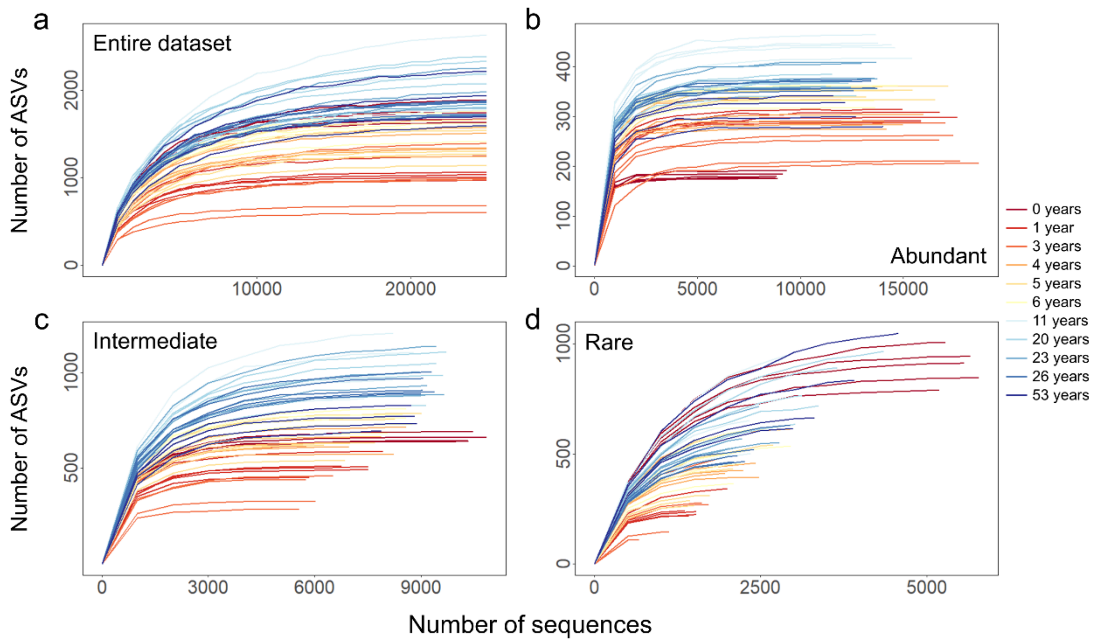

Supplementary Figure 4. Rarefaction curves of individual samples. a Entire dataset; **b** Abundant subcommunity; **c** Intermediate subcommunity; **d** Rare subcommunity. Lines with different colors indicate samples with different restoration durations.

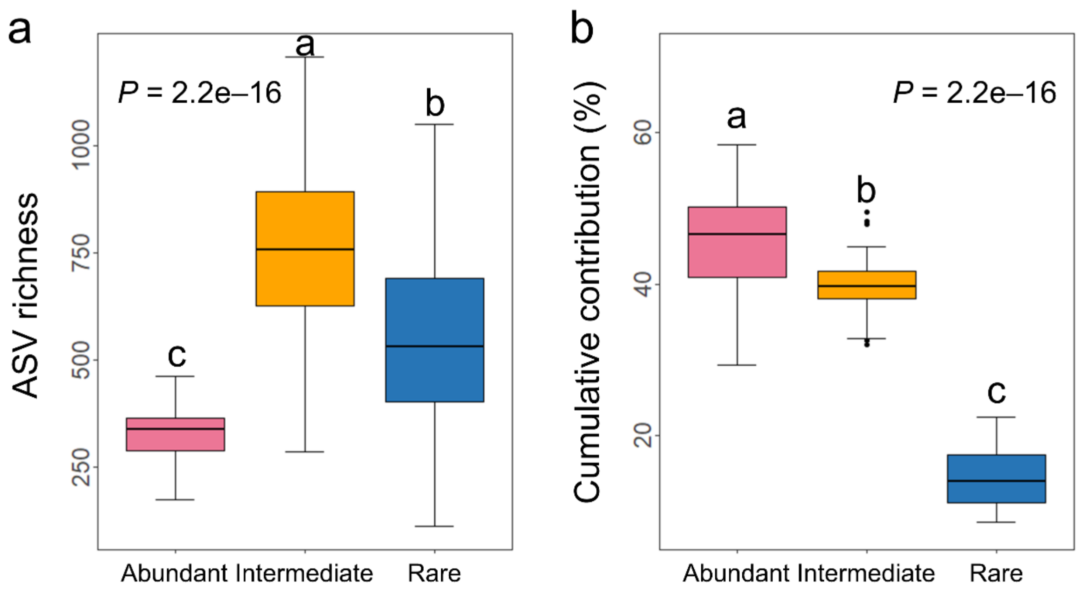

Supplementary Figure 5. ASV richness and cumulative contributions to community differences in abundant, intermediate, and rare taxa. a Boxplots of the ASV richness of abundant, intermediate, and rare taxa per sample ($n = 55$). The different letters represent significant differences determined by the Kruskal–Wallis test followed by Dunn post-hoc test (Bonferroni correction). **b** Boxplots of the cumulative contributions of abundant, intermediate, and rare taxa to pairwise composition differences ($n = 55$) using SIMPER analysis based on Bray–Curtis distance. The different letters represent significant differences determined by the Kruskal–Wallis test followed by Dunn post-hoc test (Bonferroni correction). The specific values were presented in Supplementary Table 2.

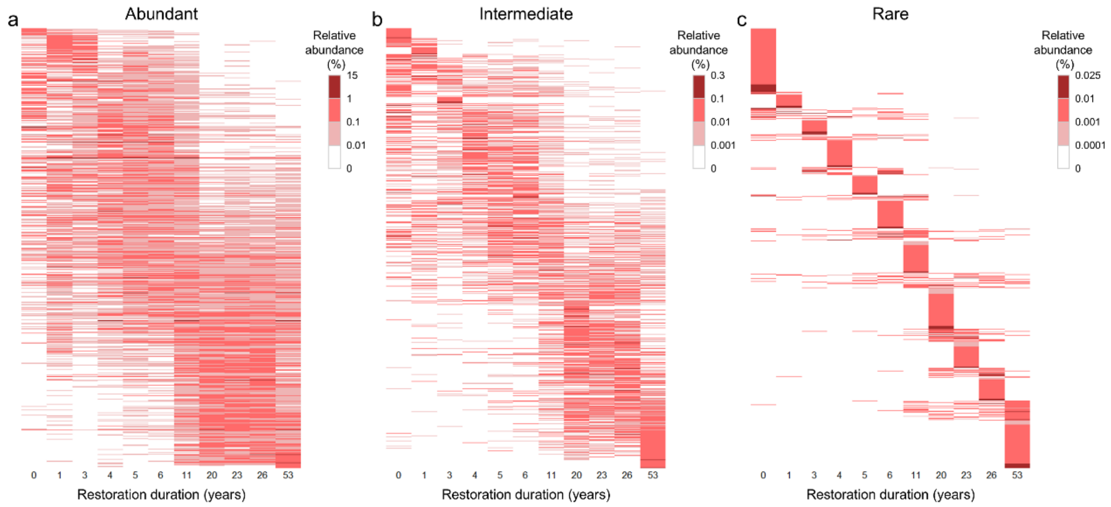

Supplementary Figure 6. Heatmaps of the relative abundance of abundant, intermediate, and rare ASVs. a–c The arrangement of relative abundance for each abundant, intermediate, and rare ASV along the 53-year restoration chronosequence. The gradient of red color intensity represents the relative abundance levels, with darker shades indicating higher abundance and lighter shades denoting lower abundance.

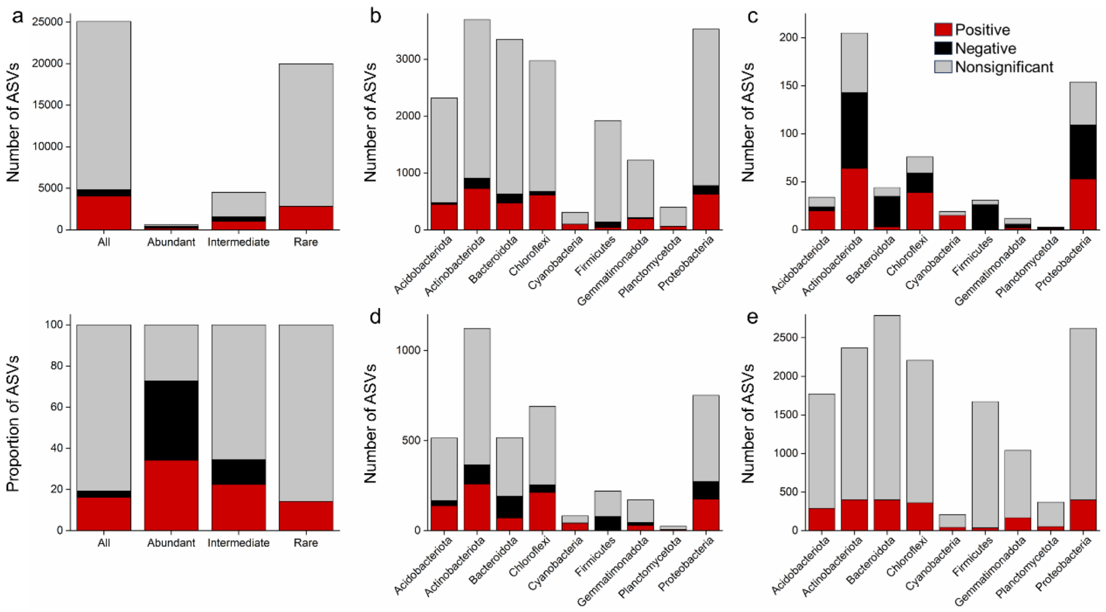

Supplementary Figure 7. Number of ASVs with significant or nonsignificant changes in relative abundance along restoration duration. a The number and proportion of ASVs with significant or insignificant changes in relative abundance along restoration duration in the entire community, as well as in the abundant, intermediate, and rare subcommunities. **b**–**e** The number of these ASVs at the phylum level in the entire community, as well as in the abundant, intermediate, and rare subcommunities. Statistically significant positive and negative ($P \leq 0.05$), and nonsignificant ($P > 0.05$) changes based on Pearson correlation are indicated by red, black, and gray, respectively.

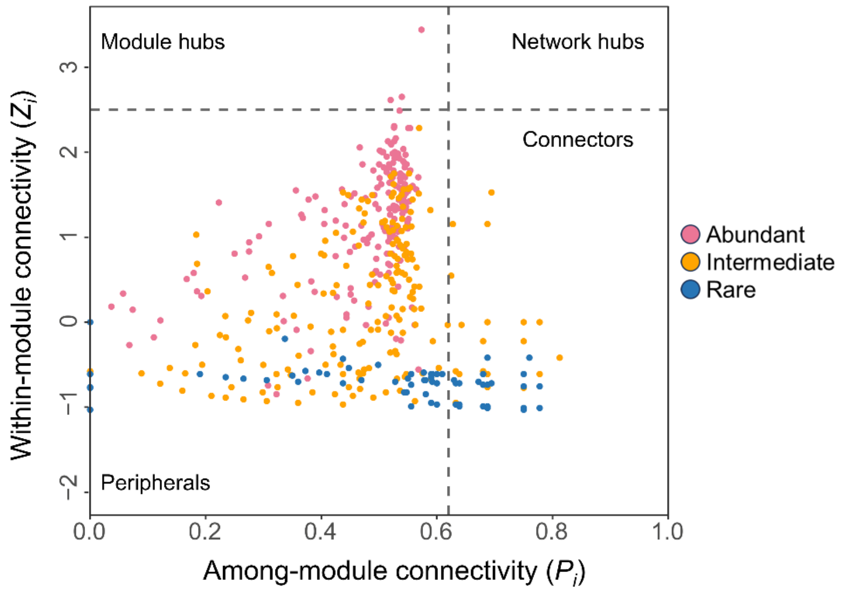

Supplementary Figure 8. Topological roles of nodes determined by their within-module connectivity and among-module connectivity. Each dot represents a node. The module hubs, connectors and network hubs are regarded as keystone nodes.

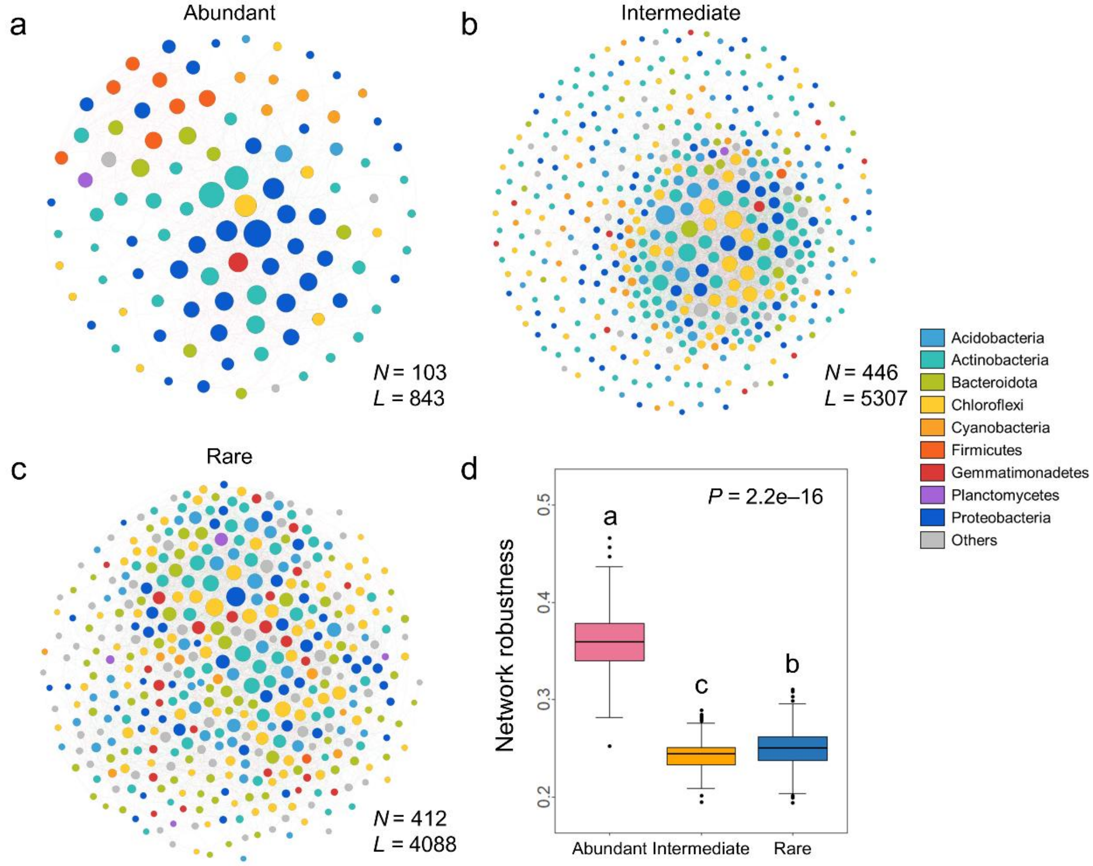

Supplementary Figure 9. Separate networks and their robustness. a–c Separate networks for abundant, intermediate, and rare taxa. *N*, the number of nodes; *L*, the number of edges. The size of each node is proportional to its degree. **d** Boxplots of the robustness of separate networks for abundant, intermediate, and rare taxa at 1000 iterations ($n = 1000$). The different letters represent significant differences determined by the Kruskal–Wallis test followed by Dunn post-hoc test (Bonferroni correction).

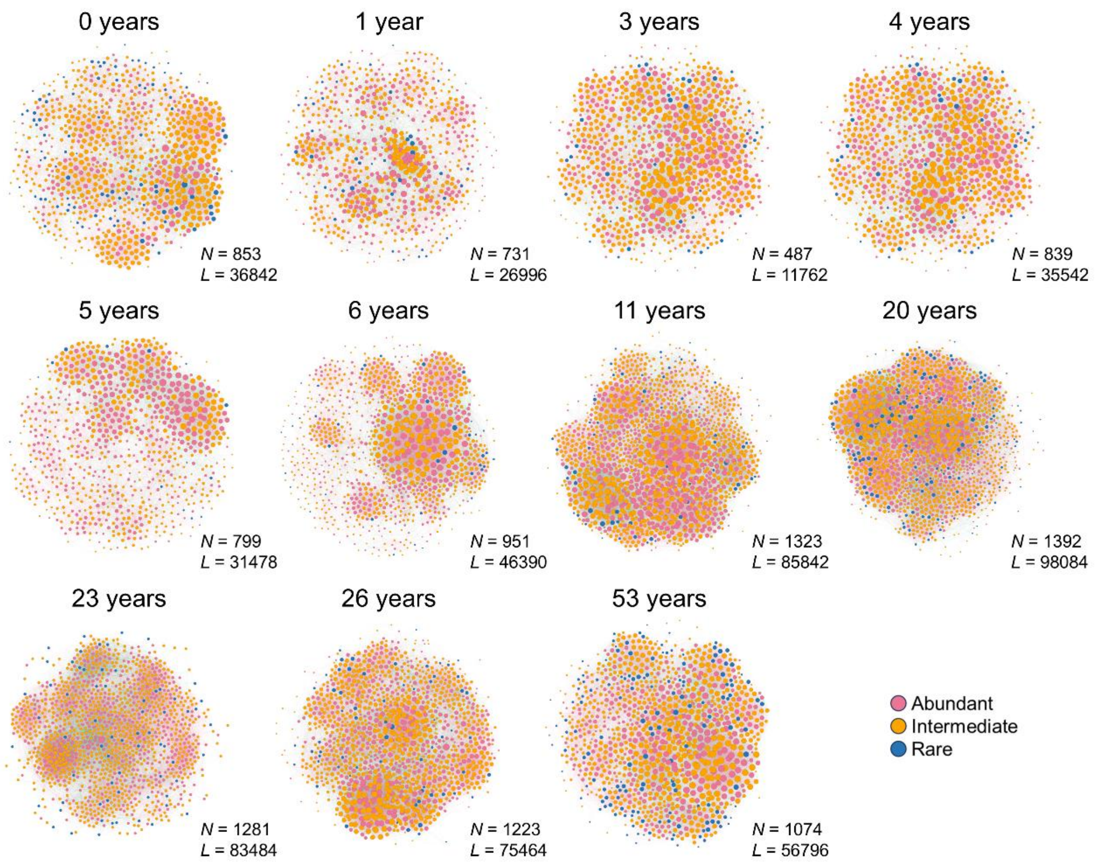

Supplementary Figure 10. Networks of abundant, intermediate, and rare taxa at each restoration stage. *N*, the number of nodes; *L*, the number of edges. The size of each node is proportional to its degree.

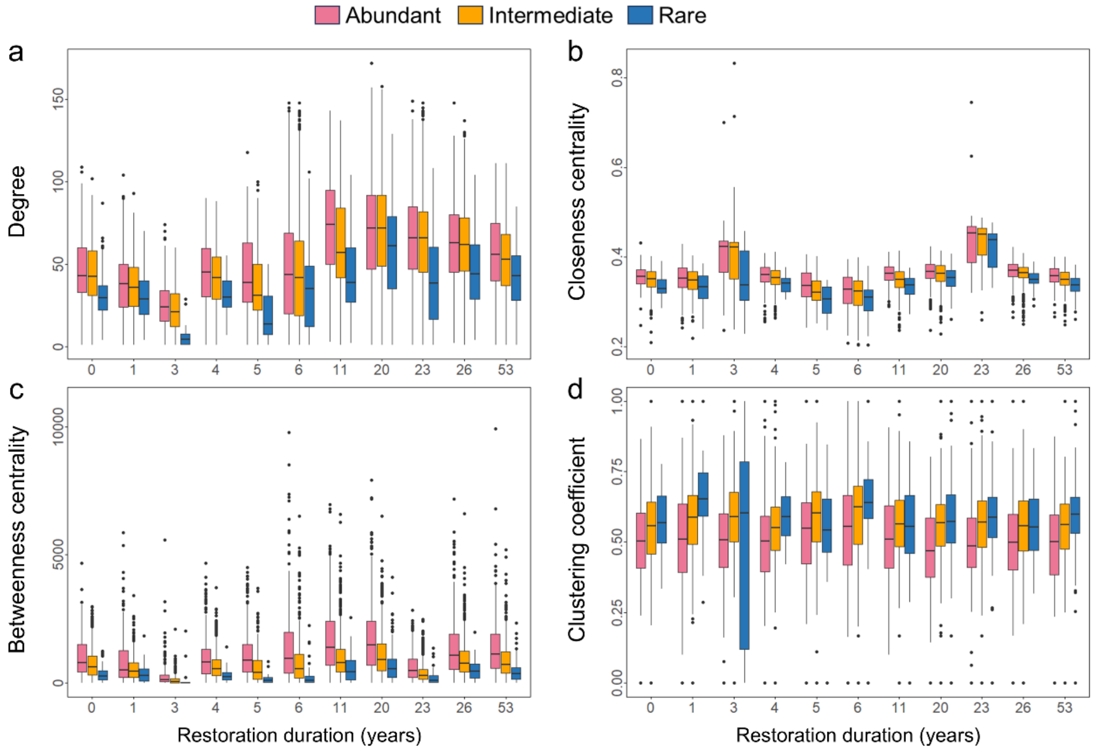

Supplementary Figure 11. Node-level topological properties of abundant, intermediate, and rare taxa in subnetworks at each restoration stage. a Degree; **b** Closeness centrality; **c** Betweenness centrality; **d** Clustering coefficient.

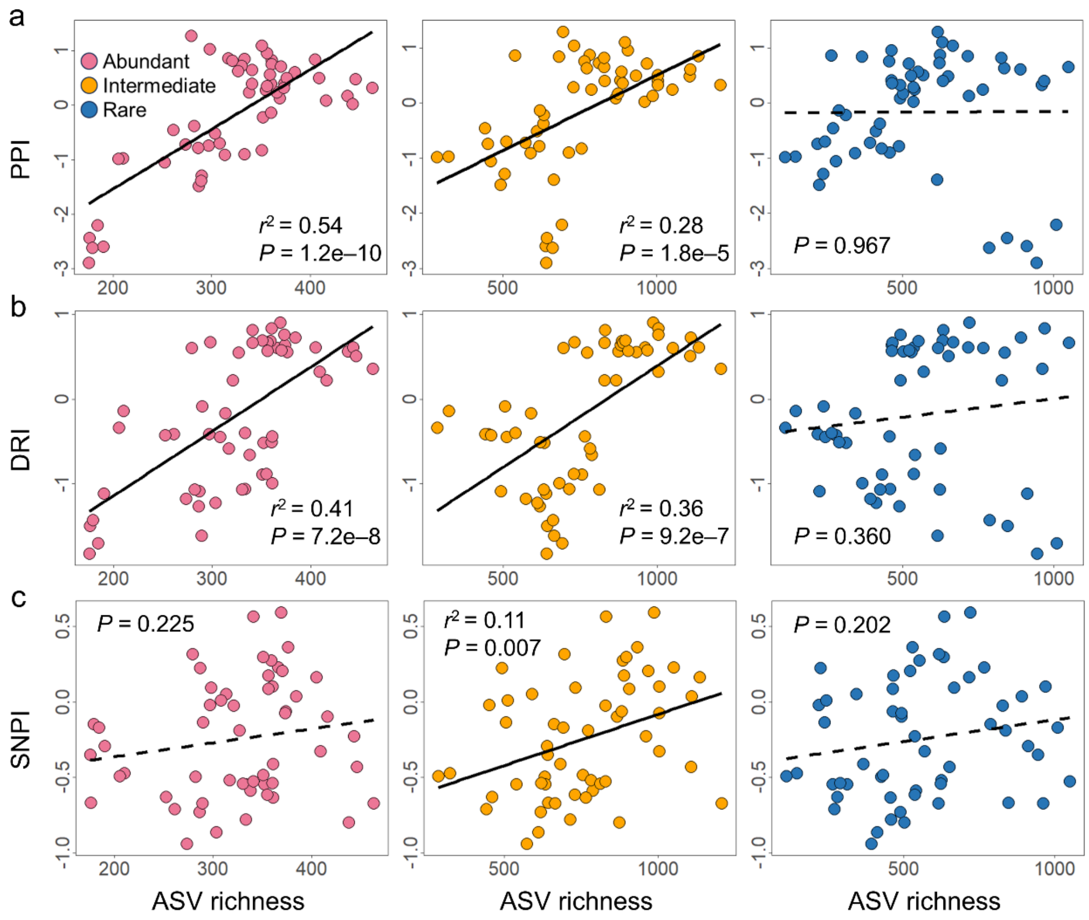

Supplementary Figure 12. Relationships between multifunctionality indices and richness of abundant, intermediate, and rare taxa. a PPI; **b** DRI; **c** SNPI. The black fitted lines are from linear regression. The solid and dotted lines represent statistically significant ($P \leq 0.05$) and nonsignificant ($P > 0.05$) relationships, respectively. PPI, plant productivity index; DRI, decomposition rate index; SNPI, soil nutrient pool index.

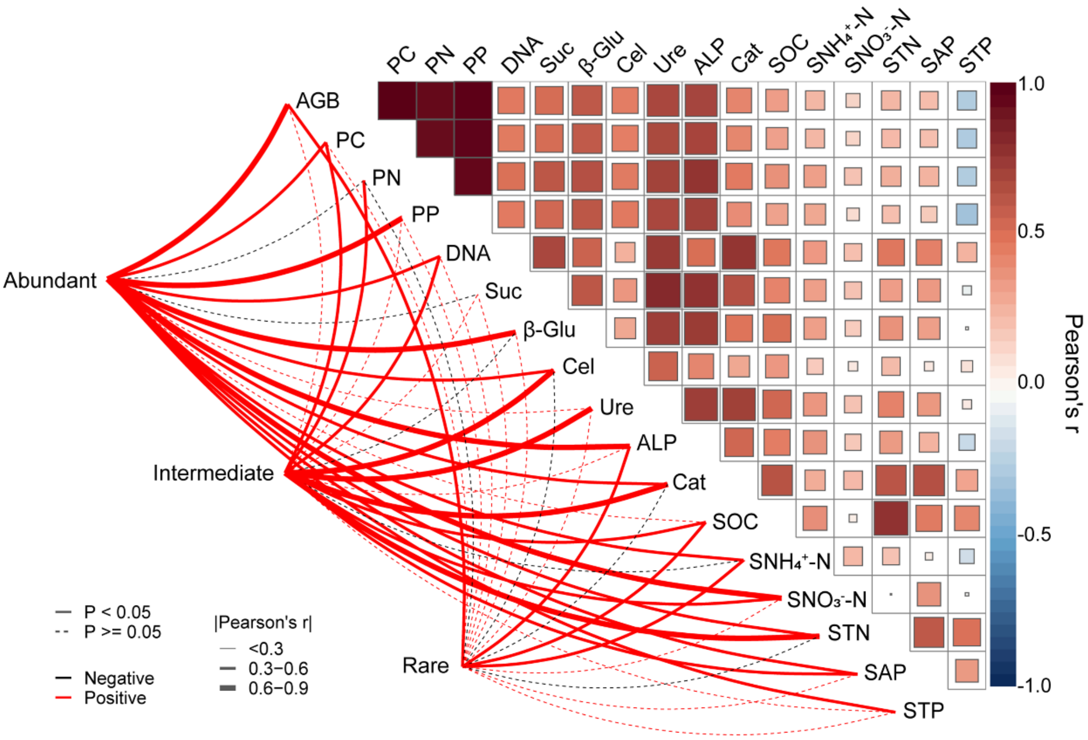

Supplementary Figure 13. Relationships of richness of abundant, intermediate, and rare taxa with individual functions. Edge width corresponds to the Pearson's r value. Positive and negative correlations are indicated by red and black edges, respectively. Pairwise correlations of these functions are displayed with a color gradient denoting Pearson's correlation coefficient. AGB, aboveground biomass; PC, plant carbon; PN, plant nitrogen; PP, plant phosphorus; DNA, soil DNA concentration; Suc, soil sucrase activity; β-Glu, soil β-glucosidase activity; Cel, soil cellulase activity; Ure, soil urease activity; ALP, soil alkaline phosphatase activity; Cat, soil catalase activity; SOC, soil organic carbon; SNH_4^+-N, soil ammonium; SNO_3^--N, soil nitrate; STN, soil total nitrogen; SAP, soil available phosphorus; STP, soil total phosphorus.

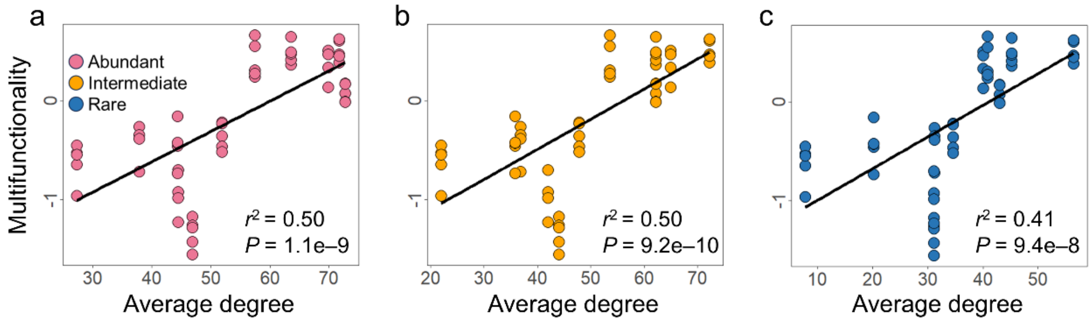

Supplementary Figure 14. Relationships between multifunctionality and average degree of abundant, intermediate, and rare taxa. a Abundant taxa; **b** Intermediate taxa; **c** Rare taxa. The average degree was derived from the subnetworks of each restoration stage. The black fitted lines are from linear regression.

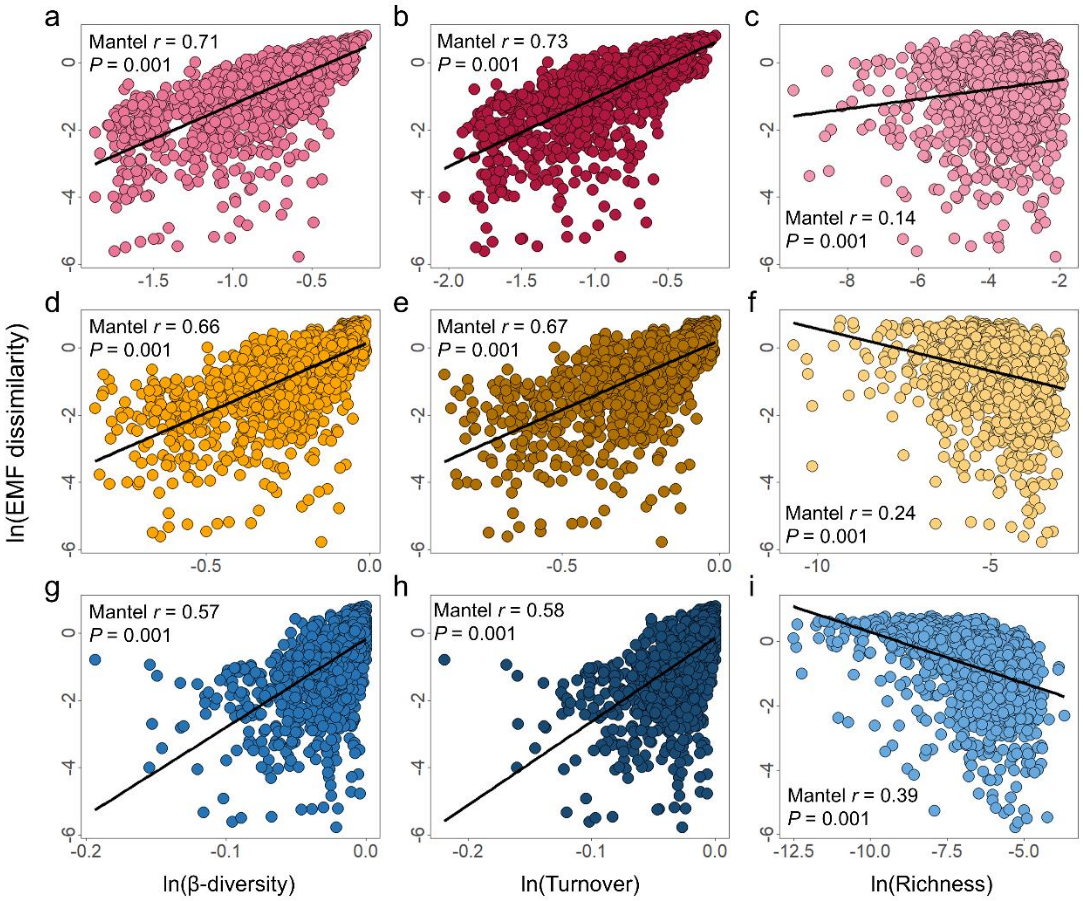

Supplementary Figure 15. Relationships between multifunctionality dissimilarity and β-diversity and its components of abundant, intermediate, and rare subcommunities based on the Mantel test. a–c Abundant taxa; **d–f** Intermediate taxa; **g–i** Rare taxa. The black fitted lines are from linear regression.

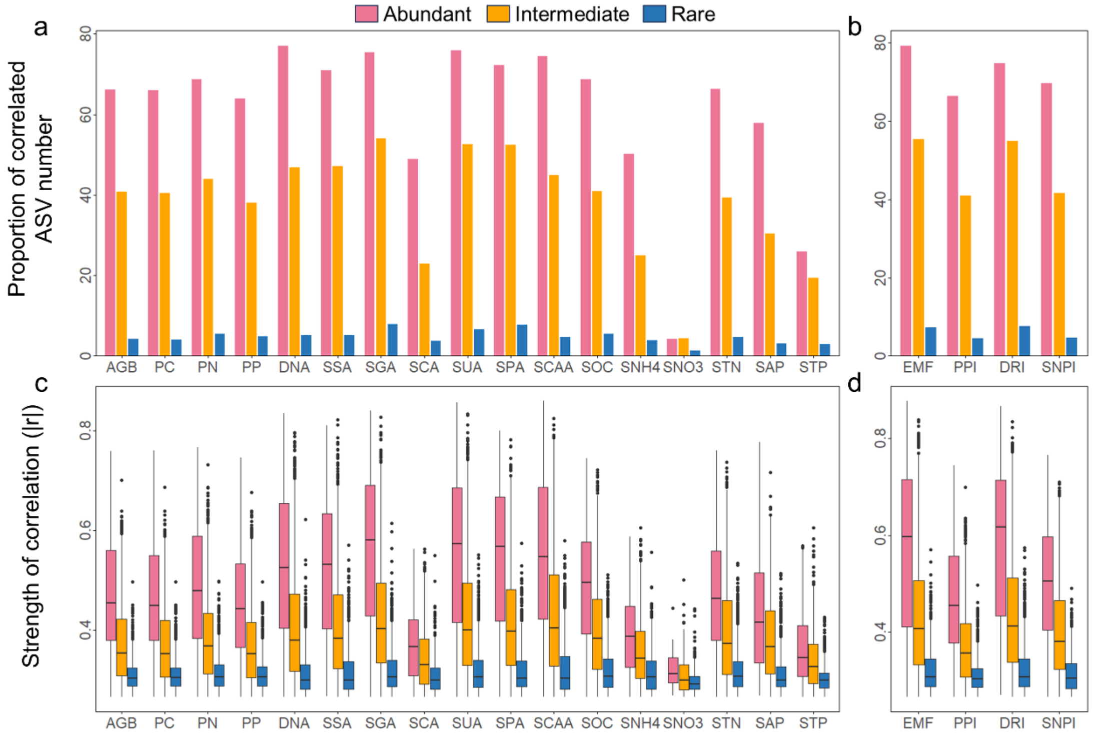

Supplementary Figure 16. Proportion and correlation strength of ASVs that were significantly associated with individual functions and multifunctionality indices for abundant, intermediate, and rare taxa. a, c Individual functions; **b, d** multifunctionality indices. AGB, aboveground biomass; PC, plant carbon; PN, plant nitrogen; PP, plant phosphorus; DNA, soil DNA concentration; Suc, soil sucrase activity; β-Glu, soil β-glucosidase activity; Cel, soil cellulase activity; Ure, soil urease activity; ALP, soil alkaline phosphatase activity; Cat, soil catalase activity; SOC, soil organic carbon; SNH_4, soil ammonium; SNO_3, soil nitrate; STN, soil total nitrogen; SAP, soil available phosphorus; STP, soil total phosphorus; EMF, ecosystem multifunctionality; PPI, plant productivity index; DRI, decomposition rate index; SNPI, soil nutrient pool index.

Supplementary Table 1. Best models for richness of abundant, intermediate and rare taxa.

Model	Abundant		Intermediate		Rare	
	r^2	AIC	r^2	AIC	r^2	AIC
Linear	0.08	623	0.19	735	0.15	752
Quadratic	0.49	592	0.52	709	0.13*	754
Logarithmic	0.41	599	0.40	718	0.04	758
Restoration model	0.62	578	0.54	707	-	-

Lower AIC values indicate a better fit of the model.

Supplementary Table 2. Cumulative contributions of abundant, intermediate, and rare taxa to pairwise composition differences based on Bray–Curtis distance.

Taxa	Time	0 years	1 year	3 years	4 years	5 years	6 years	11 years	20 years	23 years	26 years
Abundant	1 year	46.58									
	3 years	50.22	51.28								
	4 years	40.67	51.46	57.23							
	5 years	44.24	48.93	55.89	47.12						
	6 years	39.46	47.34	54.58	42.63	43.75					
	11 years	42.06	50.10	57.55	47.31	46.42	43.41				
	20 years	38.50	49.42	55.44	46.76	46.16	43.36	37.45			
	23 years	41.25	51.76	57.81	48.72	48.05	45.47	39.33	29.80		
	26 years	41.65	52.40	58.38	49.53	48.98	46.09	39.57	29.34	31.18	
	53 years	39.97	50.51	56.04	46.76	46.85	44.30	39.35	33.37	36.41	35.98
Intermediate	1 year	37.22									
	3 years	34.32	38.17								
	4 years	39.21	37.45	32.57							
	5 years	38.03	40.24	34.45	39.16						
	6 years	40.64	40.54	34.63	41.45	41.65					
	11 years	39.52	38.59	32.79	39.10	40.21	41.74				
	20 years	41.98	38.32	33.56	39.28	40.39	41.73	44.43			
	23 years	41.55	38.43	33.58	39.64	40.90	42.01	44.74	47.90		
	26 years	41.24	37.86	33.07	38.95	40.06	41.46	44.66	48.27	49.46	
	53 years	40.06	36.48	32.04	38.14	38.60	39.73	42.23	44.37	44.73	44.90
Rare	1 year	16.20									
	3 years	15.46	10.55								
	4 years	20.12	11.10	10.21							
	5 years	17.72	10.83	9.66	13.72						
	6 years	19.91	12.12	10.79	15.92	14.60					
	11 years	18.42	11.31	9.67	13.58	13.37	14.85				
	20 years	19.52	12.26	11.00	13.96	13.46	14.92	18.12			
	23 years	17.20	9.80	8.60	11.63	11.05	12.53	15.93	22.30		
	26 years	17.11	9.74	8.55	11.51	10.96	12.45	15.76	22.39	19.35	
	53 years	19.97	13.01	11.92	15.10	14.55	15.97	18.42	22.26	18.86	19.12
