# Supplementary material for: Divergent community assembly processes and multifunctionality contributions of abundant and rare soil bacteria during a 53-year restoration in the Tengger Desert, China
Source: Commun Biol. 2025 Sep 26;8:1376. doi: 10.1038/s42003-025-08764-8 (PMC12474879; doi:10.1038/s42003-025-08764-8)
Supplement: Supplementary file 2 — Reporting summary [file 42003_2025_8764_MOESM2_ESM.pdf]

nature portfolio

Corresponding author(s): Jianming Deng; Weigang Hu

Last updated by author(s): Aug 7, 2025

Reporting Summary

Nature Portfolio wishes to improve the reproducibility of the work that we publish. This form provides structure for consistency and transparency in reporting. For further information on Nature Portfolio policies, see our Editorial Policies and the Editorial Policy Checklist.

Statistics

For all statistical analyses, confirm that the following items are present in the figure legend, table legend, main text, or Methods section.

n/a	Confirmed	
☐	☒	The exact sample size (*n*) for each experimental group/condition, given as a discrete number and unit of measurement
☐	☒	A statement on whether measurements were taken from distinct samples or whether the same sample was measured repeatedly
☐	☒	The statistical test(s) used AND whether they are one- or two-sided<br>*Only common tests should be described solely by name; describe more complex techniques in the Methods section.*
☒	☐	A description of all covariates tested
☐	☒	A description of any assumptions or corrections, such as tests of normality and adjustment for multiple comparisons
☐	☒	A full description of the statistical parameters including central tendency (e.g. means) or other basic estimates (e.g. regression coefficient) AND variation (e.g. standard deviation) or associated estimates of uncertainty (e.g. confidence intervals)
☐	☒	For null hypothesis testing, the test statistic (e.g. F, t, r) with confidence intervals, effect sizes, degrees of freedom and P value noted<br>*Give P values as exact values whenever suitable.*
☒	☐	For Bayesian analysis, information on the choice of priors and Markov chain Monte Carlo settings
☒	☐	For hierarchical and complex designs, identification of the appropriate level for tests and full reporting of outcomes
☐	☒	Estimates of effect sizes (e.g. Cohen's *d*, Pearson's *r*), indicating how they were calculated

Our web collection on statistics for biologists contains articles on many of the points above.

Software and code

Policy information about availability of computer code

Data collection	No software was used.
Data analysis	Cutadapt (version 1.18), fastp (version 0.23.4), QIIME (version 2024.10), and SILVA prokaryotic database (version 138) were used to perform bioinformatic analyses. We conducted statistical analyses and figure production using R (version 4.4.1) and Gephi (version 0.9.2).

For manuscripts utilizing custom algorithms or software that are central to the research but not yet described in published literature, software must be made available to editors and reviewers. We strongly encourage code deposition in a community repository (e.g. GitHub). See the Nature Portfolio guidelines for submitting code & software for further information.

Data

Policy information about availability of data

All manuscripts must include a data availability statement. This statement should provide the following information, where applicable:

- Accession codes, unique identifiers, or web links for publicly available datasets
- A description of any restrictions on data availability
- For clinical datasets or third party data, please ensure that the statement adheres to our policy

The datasets that support the main findings of this study are publicly available on figshare [https://doi.org/10.6084/m9.figshare.26778400]. The raw bacterial sequence data have been deposited in the NCBI SRA database under accession code PRJNA877077.

Research involving human participants, their data, or biological material

Policy information about studies with human participants or human data. See also policy information about sex, gender (identity/presentation), and sexual orientation and race, ethnicity and racism.

Reporting on sex and gender	N/A
Reporting on race, ethnicity, or other socially relevant groupings	N/A
Population characteristics	N/A
Recruitment	N/A
Ethics oversight	N/A

Note that full information on the approval of the study protocol must also be provided in the manuscript.

Field-specific reporting

Please select the one below that is the best fit for your research. If you are not sure, read the appropriate sections before making your selection.

☐ Life sciences ☐ Behavioural & social sciences ☒ Ecological, evolutionary & environmental sciences

For a reference copy of the document with all sections, see nature.com/documents/nr-reporting-summary-flat.pdf

Ecological, evolutionary & environmental sciences study design

All studies must disclose on these points even when the disclosure is negative.

Study description	In this study, we surveyed soil bacterial communities along a 53-year chronosequence of desertified ecosystem restoration following straw checkerboard barrier establishment on the southern margin of the Tengger Desert in northwest China to investigate the diversity patterns, community assembly processes, and effects on multifunctionality of high-abundance and rare bacteria.
Research sample	For this study, we collected 55 soil samples from 10 plots restored by straw checkerboard barrier treatment, each with different restoration durations (i.e., 1, 3, 4, 5, 6, 11, 20, 23, 26 and 53 years), as well as from a mobile sandy land plot. A total of 55 soil samples were collected from the 11 quadrats. Physicochemical properties and genomic DNA extraction were performed on each soil sample.
Sampling strategy	In each plot, a 12 × 12 m quadrat was established under the most representative and homogeneous vegetation. Five 12-m-long transects (spaced 1.2 m apart) were arranged within each quadrat to harvest above-ground plant materials following the line-intercept protocol. In each quadrat, five 1.2 × 1.2 m subplots (spaced at least 4 m apart) were randomly selected to collect soil samples. In each subplot, 5–7 topsoil cores (0–10 cm depth) were collected randomly, and then mixed to form a single sample. After field sampling, all soil samples were taken to the laboratory and sieved (< 2 mm). Each soil sample was divided into three parts. One part was dried for the determination of physicochemical properties, another part was stored at 4 °C for measuring enzyme activities, and the last part was stored at –80 °C for DNA extraction (< 1 week).
Data collection	For field studies, the coordinates of each plot were recorded by Qingqing Hou using a portable GPS device. Qingqing Hou and Ying Sun extracted soil genomic DNA from soil using the PowerSoil DNA Isolation Kit (MO BIO Laboratories, USA). Bacterial sequence data were obtained by sequencing the V3-V4 regions of the bacterial 16S rRNA gene with primer pair 338F/806R and processed by Qingqing Hou and Abdul Manan. All the authors measured aboveground biomass and soil physical and chemical properties in Jianming Deng' laboratory.
Timing and spatial scale	For the field study, sampling took place between August and September 2017. The spatial scale from which the field data were taken is region.
Data exclusions	No data were excluded from the analyses.
Reproducibility	To aid in the reproducibility of our analyses, we provided source data to reproduce the main findings described in the manuscript through figshare [https://doi.org/10.6084/m9.figshare.26778400].
Randomization	For all study plots, the dryland subtype is arid regions based on a published classification by Pointing & Belnap (Nat. Rev. Microbiol. 10, 551-562, 2012), and the vegetation type is desert based on a published vegetation atlas of China (Chinese Academy of Sciences, 2001).
Blinding	Blinding was not relevant to this study as all samples were simply given an independent number system which had no information on either site or microcosm treatment and all data were processed and analyzed using available software with automated algorithms.

Did the study involve field work? ☒ Yes ☐ No

Field work, collection and transport

Field conditions	In the field study area, the mean elevation, mean annual temperature and mean annual precipitation are 1680 m a.s.l., 8.7 °C and 179 mm, respectively. In order to prevent desert invasion and restore desertified ecosystems, straw checkerboard barriers have been established.
Location	Plot type Duration Latitude Longitude Elevation/m<br>mobile sandy land 0 37.55 103.79 1727.00<br>Restoration 1 37.56 103.79 1733.50<br>Restoration 3 37.56 103.80 1731.00<br>Restoration 4 37.56 103.80 1734.00<br>Restoration 5 37.56 103.81 1725.00<br>Restoration 6 37.56 103.81 1721.00<br>Restoration 11 37.56 103.81 1722.00<br>Restoration 29 37.56 103.84 1719.00<br>Restoration 23 37.60 103.84 1650.00<br>Restoration 26 37.60 103.84 1654.00<br>Restoration 53 37.45 104.97 1345.00
Access & import/export	No samples were imported or exported. All samples were collected in the state-owned land which is open for scientific research. No specified permissions are required for the sampling sites, which are not natural reserve and did not involve endangered or protected species.
Disturbance	This study did not cause any environmental disturbance.

Reporting for specific materials, systems and methods

We require information from authors about some types of materials, experimental systems and methods used in many studies. Here, indicate whether each material, system or method listed is relevant to your study. If you are not sure if a list item applies to your research, read the appropriate section before selecting a response.

Materials & experimental systems

n/a	Involved in the study
☒	☐ Antibodies
☒	☐ Eukaryotic cell lines
☒	☐ Palaeontology and archaeology
☒	☐ Animals and other organisms
☒	☐ Clinical data
☒	☐ Dual use research of concern
☒	☐ Plants

Methods

n/a	Involved in the study
☒	☐ ChIP-seq
☒	☐ Flow cytometry
☒	☐ MRI-based neuroimaging

Plants

Seed stocks	N/A
Novel plant genotypes	N/A
Authentication	N/A
